# Flagella-driven motility is a target of human Paneth cell defensin activity

**DOI:** 10.1371/journal.ppat.1011200

**Published:** 2023-02-23

**Authors:** Douglas T. Akahoshi, Dean E. Natwick, Weirong Yuan, Wuyuan Lu, Sean R. Collins, Charles L. Bevins

**Affiliations:** 1 Department of Microbiology and Immunology, School of Medicine, University of California Davis, Davis, California, United States of America; 2 Department of Microbiology and Molecular Genetics, University of California Davis, Davis, California, United States of America; 3 Institute of Human Virology and Department of Biochemistry and Molecular Biology, University of Maryland School of Medicine, Baltimore, Maryland, United States of America; University of Maryland, UNITED STATES

## Abstract

In the mammalian intestine, flagellar motility can provide microbes competitive advantage, but also threatens the spatial segregation established by the host at the epithelial surface. Unlike microbicidal defensins, previous studies indicated that the protective activities of human α-defensin 6 (HD6), a peptide secreted by Paneth cells of the small intestine, resides in its remarkable ability to bind microbial surface proteins and self-assemble into protective fibers and nets. Given its ability to bind flagellin, we proposed that HD6 might be an effective inhibitor of bacterial motility. Here, we utilized advanced automated live cell fluorescence imaging to assess the effects of HD6 on actively swimming *Salmonella enterica* in real time. We found that HD6 was able to effectively restrict flagellar motility of individual bacteria. Flagellin-specific antibody, a classic inhibitor of flagellar motility that utilizes a mechanism of agglutination, lost its activity at low bacterial densities, whereas HD6 activity was not diminished. A single amino acid variant of HD6 that was able to bind flagellin, but not self-assemble, lost ability to inhibit flagellar motility. Together, these results suggest a specialized role of HD6 self-assembly into polymers in targeting and restricting flagellar motility.

## Author summary

Many microbes use flagella, whip-like organelles, to propel through their environment to meet their lifestyle requirements. In the environment of the intestinal tract, microbes can utilize flagellar motility to facilitate colonization, and for some enteric pathogens, including *Salmonella enterica*, flagella are an essential virulence factor to breach the protective mucosal barrier and gain access to the epithelial surface. Previous studies found that human α-defensin 6 (HD6), a peptide secreted by Paneth cells of the small intestine, is host-protective, not through microbicidal activity characteristic of most other defensins, but through its remarkable ability to bind bacterial surface proteins and then self-assemble into fibers and nets. Here, we utilized advanced fluorescence microscopy to assess the effects of HD6 on actively swimming *Salmonella enterica* to test the hypothesis that HD6 is an effective inhibitor of flagellar motility. We report that HD6 was able to effectively restrict flagellar motility of individual bacteria. A single amino acid variant of HD6, that like native HD6 was also able to bind flagellin but not self-assemble, lost ability to inhibit flagellar motility. Our results point to a likely key function of HD6 self-assembly into polymers in targeting and restricting flagellar motility.

## Introduction

A critically important biological boundary for host health and survival exists at the intestinal mucosa, where a complex ecosystem of microbes intimately colonizes an organ system tasked with nutrient uptake [1–3]. Here, colonizing microbes can benefit the host in many physiological functions, including nutrient acquisition [4–10]. Yet, harmful microbes can access this anatomic site to cause disease, often through a strategy of contacting and breaching the intestinal epithelium [11–13]. Effective immunity in the intestine must allow for a controlled colonization with the symbiotic microbiota, while being ever ready to protect the host from pathogens [13–18].

Colonization of the mammalian intestinal tract requires microbes to overcome many challenges, including intense competition with co-colonizers for nutrients, displacement by peristalsis, toxicity inherent to host digestive factors such as bile salts and hydrolytic enzymes, and host-derived immune effector molecules [5,19–21]. In such an environment, locomotion can afford microbes a competitive advantage by providing an ability to access nutrient-rich niches and avoid displacement or noxious microenvironments [22–24]. Many microbes achieve locomotion via flagella, whip-like organelles that can provide effective propulsion [25,26]. In addition to certain commensal microbes utilizing flagella to facilitate their intestinal colonization, numerous enteric pathogens use flagellar motility as an essential virulence factor [27]. As such, from the host’s perspective, flagella-mediated motility carries potential risk, since motility can enable microbes to breach of protective barriers responsible for spatial segregation, and even facilitate their epithelial invasion and translocation into underlying tissue [12,27]. In turn, the mammalian host has developed a repertoire of immune countermeasures to mitigate potential threat from flagellar motility, including mechanisms of flagellar detection and response by both innate and adaptive immune mechanisms [24,28–31].

Paneth cells are specialized secretory epithelial cells of the small intestine that play a crucial role in maintaining mucosal integrity and homeostasis through their secretion of a mixture of proteins and peptides that target intestinal microbes [32,33]. Through delivery of these molecules into the luminal environment, Paneth cells help mediate two interrelated functions: defend the host from enteric pathogens and maintain homeostasis with the colonizing microbiota [32–34]. A prominent secretory component of the Paneth cell are α-defensins, a group of tri-disulfide, cationic peptides with antimicrobial properties [32,35,36]. Whereas Paneth cells of some mammals express multiple α-defensin paralogs, humans express only two—human α-defensin-5 and -6 (HD5 and HD6, respectively) [32,37,38]. Like most other mammalian α-defensins, HD5 possesses direct microbicidal activity through a mechanism that targets the microbial membrane, which provides the host with both significant protection from enteric pathogens, as well an ability to reshape the composition of the microbiota [39–42]. Initially, the activity of HD6 was anticipated to mimic that of HD5. Indeed, transgenic mice engineered to express HD6 in Paneth cells gained significant protection from lethal challenge with *Salmonella enterica* serovar Typhimurium (*S*. Typhimurium) [43]. However, antimicrobial assays revealed that mature HD6 lacked the microbicidal activity of HD5, leaving open the question of how expression of this peptide protected the HD6 transgenic mice, and what might be the function of this abundant peptide in the human intestine [43,44]. The conundrum was resolved when studies showed that protection of the transgenic mice from lethal challenge with *S*. Typhimurium resulted from a microbicidal-independent mechanism that prevented bacterial invasion and translocation across the epithelium [43]. Moreover, HD6 could block *S*. Typhimurium invasion of epithelial cells *in vitro*, an activity not observed with its bactericidal counterpart HD5 [43]. The ability of HD6 to block invasion was not limited to *S*. Typhimurium, but was also observed with other diverse bacterial pathogens that employ completely different cellular invasion pathways [43,45,6]. This activity against distinct and diverse bacterial pathogens implies a broad-spectrum molecular mechanism, a characteristic feature of innate immunity.

But how is HD6 able to provide this protection? An inherent activity of HD6 stems from its striking ability to self-assemble into high-order polymers, an activity not found with either HD5, endogenous mouse α-defensins or most other α-defensins [43,45,46]. Scanning- and transmission-electron microscopy images show that the 32-residue HD6 peptide can spontaneously polymerize to form macromolecular fibrils reaching microns in length, which can then form a meshwork termed “nanonets” [43,45,46]. In addition to self-assembly, HD6 can also bind to a variety of different bacterial proteins, including flagellin, fimbriae, and invasin [43]. The proposed protective mechanism thus entails a two-step process, where there is initial (stochastic) binding of HD6 to microbial surface molecules (such as fimbrin or flagellin), which then triggers the self-assembly process that forms HD6 macromolecular fibrils and nets that entangle microbes and prevent invasion of host cells. Indeed, bacteria exposed to HD6 are observed to be agglutinated in webs of nanonets [43,45,46]. However, during the earliest stages of infection, or close to the epithelium during homeostasis, single bacteria propelled by flagellar motility might better typify encounters with HD6 at the mucosal surface as it is secreted from Paneth cells and beneath a blanket of protective mucus. However, the low bacterial concentration would preclude HD6-induced agglutination [47]. Thus, we considered that an ability of HD6 to halt flagellar motility under these conditions could be vitally important to the host.

Herein, we used live-fluorescence microscopy to assess the effects of HD6 on motile, flagellated *S*. Typhimurium in real time with high resolution. Through this methodology, we determined that HD6 is able to inhibit flagellar-driven movement of individual *S*. Typhimurium, an activity not observed with a monoclonal antibody to flagellin. Through the use of the HD6 variant (HD6^F2A^) that retains the capacity to bind flagellin, but lacks an ability to self-assemble, we found that self-assembly is necessary for HD6 to inhibit bacterial motility. Together, the findings elucidate the molecular underpinnings of an HD6 activity to inhibit flagella-driven motility, suggesting a unique role for this human α-defensin in host-microbe interactions at the intestinal mucosa.

## Results

### HD6 inhibits *S*. Typhimurium motility

**Fig 1:**
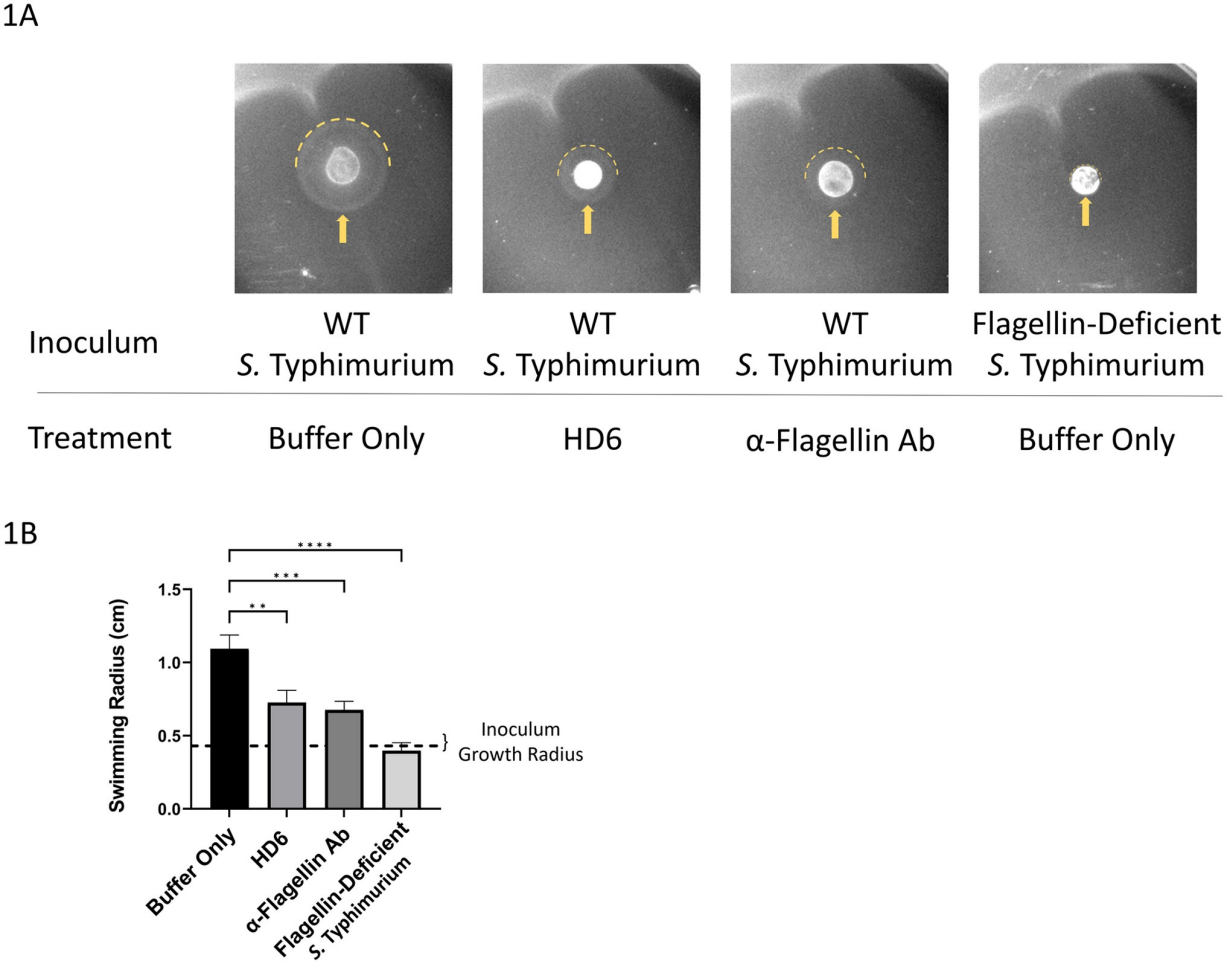
Analysis of HD6 effects on bacterial motility in semi-solid agar. (**A**) Images of semi-solid agar motility plates treated with either HD6 (30 μg), α-FliC IgG antibody (20 μg), or buffer (Tris-Maleate pH 6.4). Plates were then inoculated with either wild type (WT) *S*. Typhimurium or flagellin-deficient mutant, Δ*fliC*Δ*fljB S*. Typhimurium. After a 6.5-hour incubation at 37°C, photographic images were captured. The outer edge of the bacterial swimming halo is highlighted by the dashed semicircular yellow line and yellow arrow. Images are representative of 2 independent experiments, each with technical replicates. (**B**) Quantification of the bacterial swimming halo radius for each experimental group as in (A). The dashed horizontal line represents the radius of the inner circle, representing the inoculum growth for non-swimming bacteria, averaged for all treatments. Error bars, mean ± SD. Data were pooled from 2 independent experiments, n = 6–13 plates, and analyzed using one-way ANOVA comparing experimental groups to buffer control (**P<0.01, ***P<0.001, ****P<0.0001).

To initially assess the impact of HD6 on flagella-mediated motility, we used a semi-solid agar motility assay with a motile strain of *S*. Typhimurium. Extensive *in vitro* and *in vivo* investigations have characterized the flagellar motility of *S*. Typhimurium, making it a suitable model for our investigation [25,48]. In these assays, the presence of HD6 significantly inhibited the migration of *S*. Typhimurium, compared to migration with a buffer control (Fig 1A and 1B). Antibodies that target flagella are effective and classical inhibitors of bacterial motility [49]. A monoclonal IgG specific to *S*. Typhimurium flagellin (anti-FliC-IgG) caused a significant degree of motility inhibition to *S*. Typhimurium, comparable to that seen with HD6 treatment (Fig 1A and 1B). In these assays, neither HD6 nor anti-FliC-IgG treatment were sufficient to completely inhibit the motility of *S*. Typhimurium, when compared to a nonmotile, flagellin-deficient Δ*fliC*Δ*fljB S*. Typhimurium mutant (Fig 1A and 1B). The results from these semi-solid agar motility assays establish that HD6 is capable of inhibiting bacterial motility.

### A high-resolution live cell fluorescent microscopy assay to visualize *S*. Typhimurium motility

**Fig 2:**
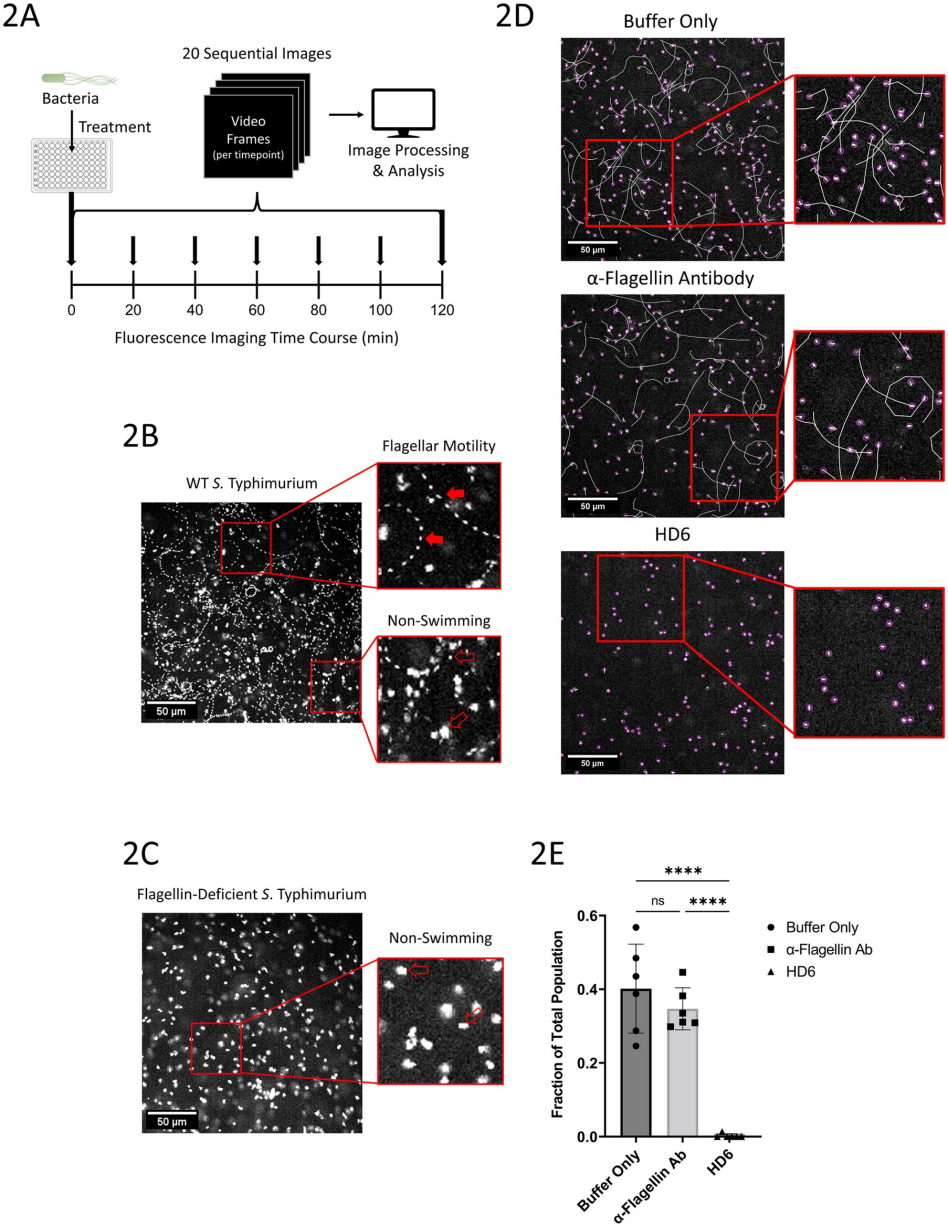
Live-fluorescence microscopy analysis of HD6 and α-FliC IgG effects on *S*. Typhimurium motility. (**A**) Schematic diagram depicting the workflow used for all live-fluorescence microscopy imaging experiments. Top Left, GFP-expressing *S*. Typhimurium are added to wells in a 96-well plate that containing different experimental treatments. Bottom, Images are captured at 20-minute intervals for each in-well position over the course of a 120-minute experiments, for a total capture of 7 timepoints. Top Right, At each timepoint, 20 sequential images are captured and Imaging data are then processed for analysis using Matlab and ImageJ. (**B**) Representative data captured at a single timepoint for GFP-expressing wild type (WT) *S*. Typhimurium in buffer. Data are presented as single superimposed image containing the 20 sequential images captured at a single timepoint, allowing for the detection of bacterial movement over time. Top Inset, Magnified image displaying an example of the characteristic pattern of *S*. Typhimurium moving via flagella driven motility (filled red arrow). Bottom Inset, Magnified image displaying examples of patterns of movement for non-swimming *S*. Typhimurium, with two examples highlighted (open red arrows). (**C**) Representative data captured at a single timepoint as in (B) for GFP-expressing flagellin-deficient mutant Δ*fliC*Δ*fljB S*. Typhimurium in buffer. Inset, Magnified image displaying examples movement for non-swimming *S*. Typhimurium, with two examples highlighted (open red arrows). No instances of swimming pattern of movement were detected with Δ*fliC*Δ*fljB S*. Typhimurium (DNS). For (**B**) and (**C**), image data are representative of 8 independent experiments with technical replicates. Scale bar, 50 μm. (**D**) Representative data for GFP-expressing wild type (WT) *S*. Typhimurium treated with either buffer alone (TOP), α-FliC IgG antibody (20 μg/ml, MIDDLE), or HD6 (10 μg/ml, BOTTOM). Images are depicted with a track overlay generated by ImageJ Trackmate. Insets, Magnified images displaying the tracks of both swimming and non-swimming *S*. Typhimurium captured using Trackmate. Track color is randomly assigned based on track ID. Image data are representative of 2 independent experiments with technical replicates. Scale bar, 50 μm. (**E**) Quantification of bacterial swimming expressed as the fraction of the total *S*. Typhimurium population that are moving via flagellar motility, as determined using the velocity track metric from Trackmate analysis. Data were pooled from 2 independent experiments, n = 6 wells, and analyzed using one-way ANOVA (Error bars, mean ± SD, ns = P>0.5, **** P<0.0001).

Whereas the agar-plate motility assay yielded a population-level assessment of bacterial motility, it did not provide the resolution necessary to visualize and study the motility of individual bacteria. Therefore, to better delineate the effects of HD6, a high-throughput, automated live cell fluorescence microscopy assay was developed to capture time-course images of the motility of GFP-expressing *S*. Typhimurium under various experimental conditions (Fig 2A). Importantly, our automated and high-throughput strategy allowed experimental parameters to be tested rapidly in parallel, which reduced experimental variability and facilitated replicate assays. Bacterial motility was visualized by capturing twenty frames in rapid succession every twenty minutes for two hours, allowing thorough assessment of movement dynamics. *S*. Typhimurium populations are known to have heterogenous expression pattern of flagellar genes, which results in given populations having both actively swimming as well as non-swimming bacteria, corresponding to flagellated bacteria and those not expressing flagellar genes, respectively [50–52]. Preliminary experiments established the imaging parameters within which treatment effects could be analyzed (DNS) and demonstrated our ability to record the movement of individual *S*. Typhimurium at a single-cell resolution (Fig 2B and S1 Video). Consistent with previous investigations on bacterial motility, the movement of individual bacteria could be categorized as either actively motile (swimming) or diffusing (Fig 2B) [52]. Diffusing bacteria (a movement coined by Furter et al. [52]) exhibited a vibration-like movement characterized by short movements in seemingly random directions resulting in minimal displacement (Fig 2B). Actively motile bacteria moved across appreciable distances in either straight, arcing, or circular paths (Fig 2B). Assays of a flagellin-deficient Δ*FliC*Δ*fljB S*. Typhimurium mutant revealed a complete absence of the actively motile population, confirming that the actively motile fraction of WT bacteria locomote via flagella-dependent swimming (Fig 2C and S2 Video). Thus, this live-fluorescence microscopy assay provided a high-throughput means to effectively observe and characterize the motility of individual bacteria.

Initial experiments using a high bacterial density (1e8 CFU/ml) showed that *S*. Typhimurium were agglutinated into large aggregates by HD6 (S1 Fig and S3–S5 Videos). Similar results were observed with *S*. Typhimurium treated with anti-FliC-IgG (S1 Fig and S4 Video). These observations are consistent with previous reports showing that both HD6 and anti-FliC-IgG are able to agglutinate *S*. Typhimurium [43,53]. Next, wells were inoculated with a reduced density of *S*. Typhimurium (5e6 CFU/ml). Under these conditions, HD6 retained its ability to inhibit the active motility of *S*. Typhimurium (S7 Video), but there was no evidence of agglutination (Fig 2D and S6 and S7 Videos). To quantify the impact of HD6 on bacterial movement, individual bacteria were tracked and measured using Trackmate, a particle tracking software plugin for ImageJ that enabled quantitation of bacterial motility based on velocity (Fig 2D) [54,55]. This analysis revealed that HD6 caused a significant reduction in the number of actively motile (swimming) *S*. Typhimurium compared to buffer treatment alone (Fig 2E). Strikingly, multiple HD6-treated wells had a complete absence of swimming *S*. Typhimurium (Fig 2D and 2E). Colony counting plate assays determined that HD6 showed no microbicidal activity in these assays (DNS), consistent with previous findings [43,45,46]. In contrast to the significant activity on bacterial motility evident with HD6, anti-FliC-IgG treatment resulted in no significant change in the population of actively motile *S*. Typhimurium compared to buffer control at this lower bacterial density (Fig 2E). Thus, despite its ability to bind flagellin and agglutinate bacteria at high density, anti-FliC-IgG was unable to inhibit the flagellar-driven motility of individual *S*. Typhimurium in these assays. These data suggested that HD6 inhibited bacterial motility by an agglutination-independent mechanism, that was distinct from inhibition of motility through anti-FliC-IgG-mediated bacterial agglutination.

**Fig 3:**
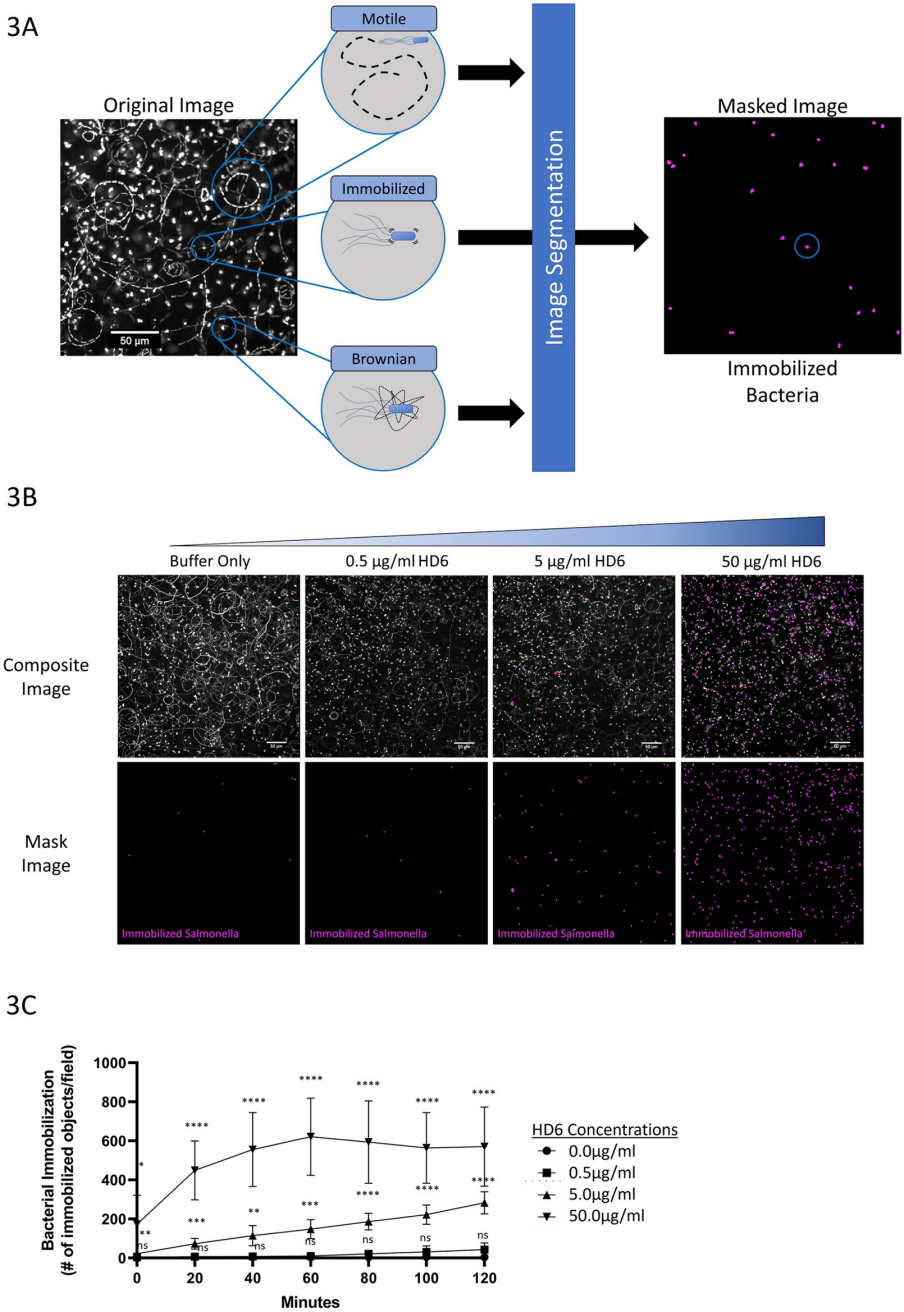
Live-fluorescence microscopy analysis of HD6-mediated *S*. Typhimurium immobilization. (**A**) Diagram depicting the masking strategy used to identify and quantify immobilized GFP-expressing *S*. Typhimurium. Left, An original superimposed image (as in Fig 2B) displaying examples of the three patterns of movement: motile, immobilized, Brownian motion. Inset circles, schematic depictions of the three patterns of movement. Right, The image mask identifies and then only displays immobilized bacteria, based on invariant spatial location in the 20 sequential images at each experimental timepoint. The resulting masked image displays the immobilized *S*. Typhimurium (red) with the image of a single bacterium highlighted (blue circle). Scale bar, 50 μm. (**B**) Representative unmasked composite data (TOP) and masked image data (BOTTOM), which depict immobilized *S*. Typhimurium observed in the presence of HD6 (0.5, 5.0, 50 μg/ml) or with buffer alone. Composite images consist of an overlay of both the original superimposed image series (white) and the image from the immobilization mask (red). The masked images (BOTTOM) display only immobilized *S*. Typhimurium (red). Data are representative of 3 independent experiments, each with technical replicates. Scale bar, 50 μm. (**C**) Quantification of *S*. Typhimurium immobilization for each experimental group as in (B). The y-axis displays the number of objects detected by in immobilized mask image for each treatment group at each experimental timepoint. The treatment groups were buffer alone (black circle), 0.5 μg/ml HD6 (black square), 5.0 μg/ml HD6 (black triangle), and 50.0 μg/ml HD6 (inverted black triangle). The data for buffer alone and 0.5μg/ml HD6 treatment groups are essentially overlapping, which obscures some of the data points. Data were pooled from 3 independent experiments, n = 7 wells. Data for HD6 treatment groups (as compared to buffer alone at each experimental timepoint) were analyzed by multiple-comparison unpaired t-test with Welch correction. Error bars, mean ± SD (ns = P>0.5, *P<0.05, **P<0.01, ***P<0.001, ****P<0.0001).

While *S*. Typhimurium treated with HD6 had a significant reduction in actively motile bacteria, there did not appear to be a corresponding increase in the diffusing population. Instead, a different population of immobilized *S*. Typhimurium were present, distinguished from the diffusing population by a complete lack of perceptible movement (Fig 2D and S7 Video). These bacteria appeared to maintain a fixed position within the field of view, often remaining in the same location across timepoints for the entire duration of the experiment. To characterize the kinetics and magnitude of this immobilization, *S*. Typhimurium were imaged upon treatment with an HD6 concentration of 0.5, 5.0, or 50 μg/ml (S9–S12 Videos). The degree of immobilization was quantified using an imaging analysis-mask capable of identifying and isolating immobilized *S*. Typhimurium population from the actively motile and diffusing populations (Fig 3A). Treatment with the 0.5 μg/ml concentration of HD6 did not yield a significant change in the proportion of immobilized bacteria compared to buffer control (Fig 3B and 3C and S9 and S10 Videos). However, at all timepoints the proportion of immobilized *S*. Typhimurium was significantly greater when treated with either 5 μg/ml or 50 μg/ml HD6 as compared to buffer control, and the proportion increased throughout the time course at these concentrations (Fig 3B and 3C and S11 and S12 Videos). Moreover, the proportion of immobilized *S*. Typhimurium at all timepoints was significantly greater after treatment with the 50 μg/ml concentration compared to treatment with HD6 at a concentration of 5 μg/ml (Fig 3B and 3C). These results demonstrate a concentration dependence in the ability of HD6 to immobilize individual bacteria.

### HD6 self-assembly is necessary for activity

**Fig 4:**
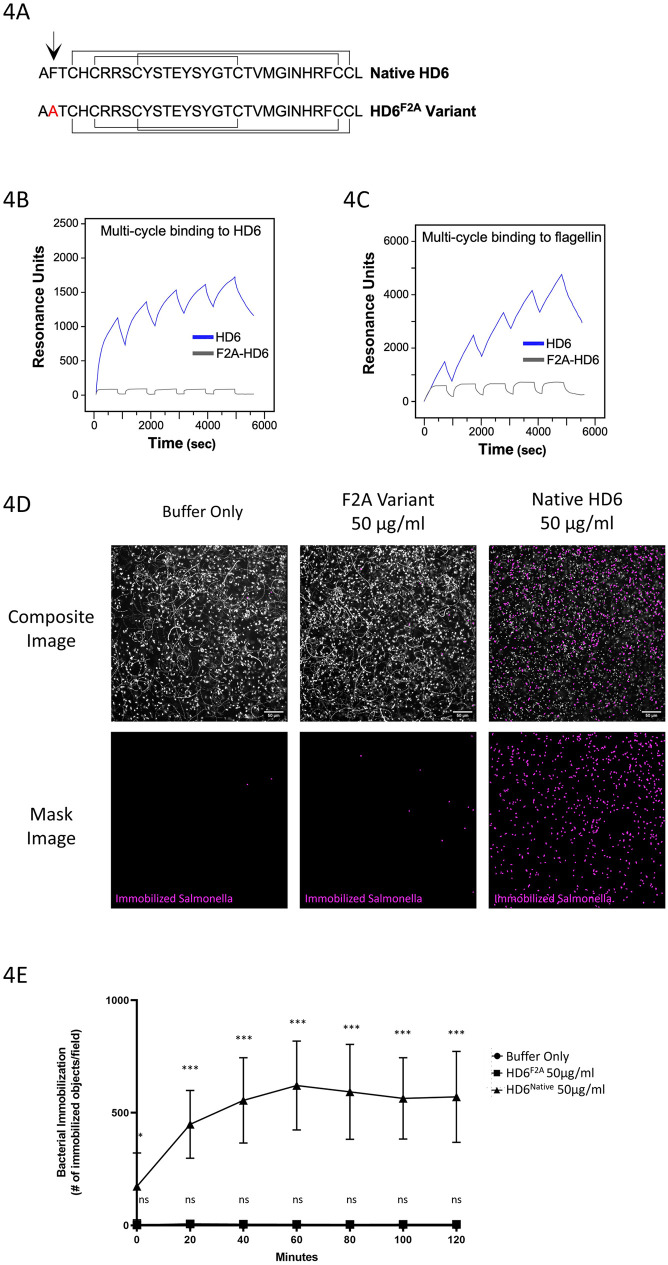
Structure-function analysis of HD6 immobilization activity. (**A**) Primary structure of native HD6 and the variant HD6^F2A^ peptides. Position two is highlighted with an arrow, with native F2 (phenylalanine) residue in black, variant A2 (alanine) in red. The connectivity of the three disulfide bonds found in HD6 and typical of α-defensins are depicted (Cys^1^-Cys^6^, Cys^2^-Cys^4^, Cys^3^-Cys^5^). (**B, C**) Surface plasmon resonance (SPR) analysis of binding and self-association. (**B**) Graph showing multi-cycle SPR results of HD6^Native^ or HD6^F2A^ binding to immobilized HD6^Native^. The SPR biosensor presented HD6 and the HD6 (blue) or HD6^F2A^ analytes traversed the biosensors at 20μl/min. Each 15 min cycle included a binding period of 12.5 min, followed by a wash period of approximately 2.5 min. (**C**) Graph showing multi-cycle SPR results of HD6^Native^ or HD6^F2A^ binding to immobilized flagellin. Analysis as in (B) except that the SPR biosensor presented flagellin isolate from *S*. Typhimurium. (**D**) Live-fluorescence microscopy analysis of *S*. Typhimurium immobilization upon treatment with either HD6 or HD6^F2A^. Representative unmasked composite data (TOP) and masked image data (BOTTOM) as in Fig 4B, which depict immobilized S. Typhimurium observed in the presence of HD6 (50 μg/ml), HD6^F2A^ (50 μg/ml) or with buffer alone. Composite images consist of an overlay of both the original superimposed image series (white) and the image from the immobilization mask (red). The masked images (BOTTOM) display only immobilized *S*. Typhimurium (red). Data are representative of 3 independent experiments with technical replicates. Scale bar, 50 μm. (**E**) Quantification of *S*. Typhimurium immobilization for each experimental group as in (D). The y-axis displays the number of objects detected by in immobilized mask image for each treatment group at each experimental timepoint. The treatment groups were buffer alone (black circle), HD6^F2A^ (50.0 μg/ml, black square), and HD6^Native^ (50.0 μg/ml, black triangle). The data for buffer alone and HD6^F2A^ treatment groups are essentially overlapping, which obscures individual data points. The data for HD6^Native^ are from Fig 4C, which was performed on the same plate. The data were pooled from 3 independent experiments, n = 7 wells. Data for HD6 and HD6^F2A^ treatment groups (as compared to buffer alone at each experimental timepoint) were analyzed by multiple-comparison unpaired t-test with Welch correction. Error bars, mean ± SD (ns = P>0.5, *P<0.05, ***P<0.001).

Previous work by our group and others showed that HD6 self-assembles into higher order polymers, which appear to create a meshwork of fibrils termed “nano-nets” that are visible through scanning- and transmission-electron microscopy [43,45,46]. Chairatana et al. found that the F2 residue in HD6 is critical for self-assembly and a HD6^F2A^ variant is unable to form fibrils or agglutinate bacteria (Fig 4A) [45]. Using multicycle-surface plasmon resonance (multicycle-SPR) to analyze the binding kinetics of HD6 and the HD6^F2A^ variant to immobilized HD6, we confirmed that HD6^F2A^ was unable to self-assemble into the higher order polymers seen with native HD6 (Fig 4B). Next, we used multicycle-SPR to analyze binding of HD6 and the HD6^F2A^ variant to immobilized flagellin isolated from *S*. Typhimurium. We found that HD6^F2A^ was able to reversibly bind to flagellin, but subsequently lacked the ability to self-assemble as seen with native HD6 (Fig 4C). Thus, these data indicated that like HD6, the HD6^F2A^ variant can bind to flagellin, but unlike HD6 was unable to self-assemble into higher-order structures.

To test if HD6-mediated immobilization of *S*. Typhimurium is dependent on its ability to self-assemble, we used our live cell microscopy assay to assess bacterial motility in the presence of HD6^F2A^ (Fig 4D and S13–S16 Videos). Quantification of bacterial immobilization revealed that treatment of *S*. Typhimurium with HD6^F2A^ had no significant effect on *S*. Typhimurium motility compared to the buffer control, even at the higher concentration of 50 μg/ml (Fig 4E). When compared to the bacterial immobilization caused by treatment with native HD6, these results indicate that the ability of HD6 to self-assemble is necessary for it to immobilize *S*. Typhimurium.

## Discussion

In the mammalian intestinal tract, many microbes use flagella-driven motility to effectively compete for colonization in this challenging environment, including some enteric pathogens that use flagella as an instrument of virulence [12,22,23,27,56]. A noteworthy feature of the immune system of mammals is the multiple overlapping mechanisms for recognition and response to flagellin, the primary protein component of flagella [24,28–30], which highlights a vital importance of targeting flagella to maintain homeostatic balance in host-microbe interactions. In this work, we asked if HD6 can interact with motile *S*. Typhimurium and inhibit flagellar motility. We developed a high-throughput, automated live cell fluorescence microscopy assay that allowed us to visualize and track the movement of individual bacteria under controlled experimental conditions. Using this approach, we demonstrated that HD6 inhibits the swimming motility of *S*. Typhimurium. Analysis of a single residue variant of HD6 revealed that the ability to bind flagellin alone is not sufficient to inhibit motility, but rather that an ability to self-assemble is also necessary. Furthermore, the inhibition of flagellar motility is distinct from flagellin-specific antibody, the classic inhibitor of flagellar motility that utilizes agglutination as a mechanism to halt movement. In assays using low bacterial densities, the flagellin-specific antibody lost its immobilization capability, whereas HD6 activity on individual swimming *S*. Typhimurium was not diminished. Collectively, our data support a model where HD6 can bind to the flagella of individual bacteria and utilize a self-assembly process that ultimately arrests movement and renders the bacterium immobilized.

Spatial segregation of the microbial-colonized lumen from the epithelium is necessary at mucosal sites for the host to maintain a balance between the flourishing microbiota and a healthy mucosa [5,13,18,57,58]. However, this homeostatic spatial segregation is threatened by flagellated microbes, because of their ability to travel through mucus and gain close proximity to the epithelium [22,52,59]. A site likely critical for the host to limit microbial access is the base of the small intestinal crypts, where stem cells that renew the intestinal epithelium reside [60]. Paneth cells are located immediately adjacent to this vulnerable stem cell niche, and so ideally positioned to secrete molecules to thwart microbial trespassers [32,33,60]. As one of the two α-defensins constitutively expressed by human Paneth cells, HD6 secreted into the crypt could immobilize and restrict the movement of individual flagellar-propelled microbes that had traversed mucus to access the crypt microenvironment.

The distinct mechanism of HD6 activity represents a significant departure from the typical microbicidal activity seen with its counterparts in the α-defensin family [36,43–45,61]. Most microbicidal α-defensin have a high degree of sequence variation from their orthologs, likely due to the biophysical properties responsible for membrane-directed activity being tolerant to amino acid substitutions [61]. In contrast to this majority of α-defensins, HD6 in its mature and folded confirmation, at concentrations 20-fold higher than those used in the current study, lacks demonstrable microbicidal activity *in vitro* [43–45,62]. Also special to HD6 is that it shares near sequence identity with its non-human primate orthologs (S2 Fig). Given that HD6 has a unique mechanism whereby it self-assembles into oligomers to prevent microbial invasion of the intestinal epithelium, the high conservation of sequence may reflect requirements for its ability to self-assemble. Chairatana and Nolan demonstrated that HD6 self-assembly is sensitive to mutation, with perturbation hydrophobic residues F2, I22, V25 and F29 preventing self-assembly and attenuating biological activity [45]. They reported that an F2A mutation, in particular, does not form fibrils nor prevent bacterial invasion of epithelial cells, consistent with the SPR findings and attenuation of biological activity we report here.

Although HD6 is a remarkable outlier within the α-defensin family, we propose that it is part of an integral arm of the innate immune system tasked with maintaining mucosal barrier integrity through recognition and inhibition of flagellar motility. In addition to molecular sensors that detect flagellin, including Toll-like receptor 5, nucleotide-binding domain leucine-rich repeat (NLR)-C4, and NLR family, apoptosis inhibitory protein (NAIP)-5 and -6 [28,63,64], an effector protein in the intestine is LYPD8, a 25 KDa colonocyte-derived protein capable of inhibiting bacterial flagellar motility by binding to a non-flagellin part of the flagellum [65,66]. In addition, akin to the action of flagellin-specific antibody, ZG16 is a lectin produced by goblet cells that can inhibit bacterial motility through a mechanism of agglutination [67]. Together, this evidence of redundancy employed by the host to recognize and inhibit microbial motility suggests these specialized effector peptides and proteins target a critical function not required for microbial replication. As such, this may highlight the value of defense strategy aimed at inhibiting a specific microbial behavior, instead of microbial eradication, which in turn would promote selection for resistance and lead to reciprocal antagonistic adaptation, the so-called "Red Queen effect" [68,69]. A non-sterilizing immune strategy targeting flagellar motility could exert host-beneficial influence not only on the behavior of pathogenic microbes, but also on members of the colonizing microbiota.

## Methods

### Bacterial cultures

Cultures of *S*. Typhimurium (S1 Table), strain IR715, were incubated in 5ml of Miller’s Luria Broth (BD 248510) under aerobic conditions at 37°C with shaking. *S*. Typhimurium containing the GFP plasmid (pDwb JK1128) were grown with carbenicillin (50 μg/ml). Cultures of Δ*fliC*Δ*fljB S*. Typhimurium (SPN313) were additionally supplemented with kanamycin (30 μg/ml). Subcultures used in microscopy and semi-solid swimming experiments were created by inoculating Miller’s Luria broth (5 ml) with the overnight culture (50 μl) and the incubating at 37°C with shaking until the broth had an OD_600_ of 0.2–0.4 (~2 hours).

To create working dilutions of bacteria, the subcultures of *S*. Typhimurium were spun at 4,000 RPM for 5 minutes in 1.5ml microcentrifuge tubes. After the centrifugation, the media supernatant was decanted, and the pellet was resuspended in sterile Tris-Maleate buffer (50mM, pH 6.4), with the volume adjusted to yield the desired bacterial concentration. In microscopy experiments requiring multiple different bacterial concentrations, serial dilutions in Tris-Maleate buffer (50mM, pH 6.4) were prepared. During the process resuspension and dilution of *S*. Typhimurium, mixing utilized gentle pipetting with care to avoid mechanical sheering of flagella from the bacteria.

### Peptides and antibody

HD6^Native^ and HD6^F2A^ were synthesized, folded, verified, and lyophilized as described previously [70,71]. Stock solutions of HD6 peptides were made by dissolving lyophilized peptides in HPLC-grade H_2_O to a concentration of 1 mg/ml. For long term storage, HD6 peptide stock solutions were aliquoted into microcentrifuge tubes, snap frozen, and stored at -80°C.

The antibody used for the agglutination microscopy was a monoclonal mouse IgG1 specific for the FliC of *S*. Typhimurium, clone X5A12 (InvivoGen, San Diego, CA, Cat# mabg-flic). Stock solutions were created by dissolving lyophilized antibody in HPLC grade H_2_O. Antibody stocks were aliquoted into microcentrifuge tubes, snap frozen, and stored at -80°C.

### Semi-solid agar motility assay

Cultured *S*. Typhimurium was suspended in Tris-Maleate buffer (50 mM, pH 6.4) at 1e7 CFU/ml. The semi-solid agar plates were composed of Tris-Maleate buffer (50 mM, pH 6.4), 0.2% w/v casamino acids (BD bacto-tryptone 211705), and 0.3% w/v agar (BD bacto-agar 244520), and prepared the day prior to the experiment. To the center each plate, an aliquot (20μl) was added of either HD6, mouse IgG1 antibody, or Tris-Maleate buffer (50 mM, pH 6.4, as vehicle control). Next, the center of each plate was inoculated with an aliquot (3 μl) of the *S*. Typhimurium suspension. Plates were then incubated at 37°C for 6.5 hours. After incubation, the plates were photographed using a gel imager (UVP, Upland, CA, BioDoc-It Imaging system). The bacterial motility/swimming parameters were assessed for each plate using ImageJ. Thus, for each image, a circular mask of swimming bacteria was generated using the “Threshold” and “Find Edges” tools. From this mask, the diameter of the circle of swimming bacteria was then measured.

### Live-fluorescence microscopy

Timelapse fluorescence microscopy of live bacteria was performed using a Nikon Ti-E inverted fluorescence microscope at 37°C. Microscopy experiments were performed using a MATLAB interface for Micromanager to enable custom, fully automated imaging. All images were acquired via epifluorescence illumination using a 20x Nikon apochromat 0.75 NA objective, the X-Cite Xylis LED illumination system, and an Andor Zyla 4.2 sCMOS camera. Experimental assays utilized Cellvis 96-well glass bottom plates (Cellvis, Mountain View, CA). Prior to imaging, buffer and peptide solutions were added to each well and allowed to equilibrate to 37°C in the microscopy chamber for ~30 minutes. After equilibration, each well was inoculated with the working bacterial suspension containing GFP-expressing *S*. Typhimurium immediately prior to the start of the experiment. Time course experiments spanned 120 minutes, and entailed 20 images acquired in rapid succession at 20-minute intervals for each experimental well (T = 0, 20, 40, 60, 80, 100, and 120 minutes). Exposure times were either 100 milliseconds or 300 milliseconds, for short and long exposure experiments, respectively.

### Agglutination assay

Prior to imaging, bacterial suspensions containing 5e8 CFU/ml of GFP-expressing *S*. Typhimurium were incubated with either HD6 (50 μg/ml), α-FliC antibody (100 μg/ml), or Tris-Maleate buffer (50mM, pH 6.8) at 37°C in microcentrifuge tubes (0.5 μl) to allow agglutination. After 30 minutes, 20 μl of this mixture was added to the microscopy plate wells containing to 80 μl of Tris-Maleate buffer (50mM, pH 6.8) equilibrated to 37°C. The final concentrations at the time of imaging were 1e8 CFU/ml GFP-expressing *S*. Typhimurium and either 10 μg/ml of HD6 or 20 μg/ml of antibody.

### Bacterial tracking assay

To observe the effects on motility of either HD6 or α-FliC antibody at a lower *S*. Typhimurium density, wells containing 5e6 CFU/ml of GFP-expressing *S*. Typhimurium and either HD6 (10 μg/ml) or of monoclonal antibody (20 μg/ml) were analyzed. An equal volume of Tris-Maleate buffer (50mM, pH 6.8) was used as a control. Experimental wells containing solely 5e6 CFU/ml GFP-expressing *S*. Typhimurium did not undergo the 30-minute preincubation step. Imaging was conducted over the course of 120 minutes in the same manner as described above.

### Immobilization assay

To observe concentration dependence of HD6-mediated bacterial immobilization, wells containing 1e7CFU/ml GFP-expressing *S*. Typhimurium were treated with 0.5, 5.0, and 50.0 μg/ml of native HD6 or HD6^F2A^ diluted in Tris-Maleate buffer (50 mM, pH 6.4) buffer. An equal volume of Tris-Maleate buffer (50mM, pH 6.8) served as a control. Imaging was conducted over the course of 120 minutes as described above.

### Image segmentation

Image processing and segmentation to identify cells for immobilization quantification were performed in MATLAB. First, background subtraction was performed on each frame to remove out-of-focus cells using the MATLAB “imopen” function with a disk-shaped structuring element and radius of 20 pixels (~13.2 microns). Next, sharpening was applied to each frame using "unsharp masking" to enhance cell edges. A single pixel intensity threshold was then determined both for each well and timepoint using the first frame of each timepoint as a reference. Accordingly, following background subtraction and sharpening of the reference frame, a crude background mask was generated by masking pixels in the 98^th^ percentile of intensity (empirically determined to generally include only bright cell pixels), applying a slight dilation using a disk-shaped structuring element and radius of 1 pixels (~0.66 microns), and then taking the complement of the mask using the MATLAB “imcomplement” function. The crude background mask was applied to the reference frame, and a final intensity threshold was determined based on the 99^th^ percentile of the remaining background pixels. For each frame of the well at the given timepoint, and following background subtraction and sharpening, cell masks were generated by applying the calculated intensity threshold, and followed by removal of small non-cell objects (< 3^2^ microns in area) using the MATLAB "bwareaopen” function.

### Immobilization analysis

A custom strategy was used to identify immobilized objects at each timepoint. For each twenty-frame time-course, the cell masks at frames 1, 10, and 20 were added together to create a composite image with a maximum value of "3" at locations where cells were present in all three frames. Objects in the composite image containing at least 50% of pixels with values of 3 were considered immobilized and were retained in the immobilization mask for that timepoint. All other objects were removed.

After an immobilization mask was created at each timepoint, immobilized objects were quantified using ImageJ. The number of immobilized objects was measured using the ImageJ “analyze particles” function. The total area of immobilized bacteria was calculated using the ImageJ “measure” function, which determines the number of pixels containing a positive signal.

### Motility analysis

The ImageJ plugin Trackmate was used to track the movement of bacteria within the well and obtain data on their movement [54,55]. Movement tracking was performed on wells containing a 5e6 CFU/ml density of *S*. Typhimurium at the 60-minute timepoint.

Raw videos were analyzed with Trackmate. The DoG detector was used to identify individual bacteria within the well. For all samples in a given experiment, the DoG detector parameters were as follows: estimated object diameter of 5, quality threshold of 3, and both pre-processing with median filter and sub-pixel localization turned on. Once bacteria were detected, the Trackmate "Kalman Tracker" was used to link the positions of the same bacteria across different frames of the video, thus producing a track corresponding to its movement. The Kalman Tracker parameters used for all samples within a given experiment were as follows: initial search radius of 25, search radius of 25, and max frame gap of 2. When necessary, incorrect spot detection and spot linking were manually corrected.

Once correct tracking of the bacteria was achieved, measurements of individual tracks were collected. These metrics included: total track length, track mean and median velocity, track duration, and total track displacement. We observed that incorrect tracking often produced a track with a low track duration. Thus, tracks with a track duration of less than or equal to 2 were omitted from the dataset.

Analysis of the track data was performed using Graphpad Prism. In order to differentiate between swimming and non-swimming bacteria, the median velocity metric was chosen to analyze the tracks. The velocity was calculated by taking the total distance travelled for a track, and then dividing by the total duration of the track, thus normalizing for variations in the duration of tracks. To create a threshold to identify bacteria that are not swimming, tracking was performed on wells containing 5e6 CFU/ml Δ*fliC*Δ*fljB S*. Typhimurium. These bacteria lack flagella, so any movement observed is due to Brownian motion. The median velocities of these tracks were pooled, and the maximum value was chosen as a threshold for the non-swimming bacteria. This threshold was applied to experimental groups to generate a ratio of swimming to non-swimming bacteria.

### Surface plasmon resonance

Surface plasmon resonance studies were performed as described previously [43]. Amine coupling was used to ligate either HD6 or flagellin to individual flow cells on a CM5 sensor chip. The running buffer (pH 7.4) contained: 10 mM HEPES, 150 mM NaCl, 3 mM EDTA, and surfactant P20 (0.005%). The multi-cycle experiments were performed with an analyte flow rate of 20 μl/min for 12.5 min in each cycle. This was followed by a 2.5 min interval during which the flow cells were perfused by analyte-free buffer until the sample loop had acquired the next 250 μl aliquot of analyte solution. The final analyte injection was in "kinetic mode", with a 10 min dissociation period.

## Supporting information

S1 Fig Live-fluorescence microscopy analysis of α-Flagellin Ab- and HD6-mediated *S*. Typhimurium agglutination. (**A)** Representative unmasked composite data depicting agglutinated *S*. Typhimurium at high densities (1e8 CFU/ml) in the presence of either 20 μg/ml α-Flagellin Ab (top row) or 10 μg/ml HD6. Areas of transiently higher bacterial density (open red arrow) and high-density bacterial aggregates (closed red arrow) are highlighted as examples of agglutination occurring. Data are representative of 2 independent experiments, each with technical replicates. Scale bar, 50 μm.
(TIF)

S2 Fig Primary amino acid comparison of HD5 and HD6 orthologs. Highlighted with YELLOW are sequence variant residues for the HD5 and HD6 orthologs, respectively. Shown in RED are the cysteine residues that participate in three intramolecular disulfide bonds (Cys^1^-Cys^6^, Cys^2^-Cys^4^, Cys^3^-Cys^5^). Highlighted with GRAY in the top consensus row are the residues identified as key for structure-function of HD6 and HD5, respectively (Chairatana and Nolan 2014; Rajabi et al. 2012). The column at the left notes the percent of residue identity for HD6 and HD5 orthologs, respectively. Within each group of orthologs, the top cluster includes great apes and the bottom Old World monkeys. Accession numbers for HD6 ortholog sequences are: Human (*Homo sapiens*), AAC50382; Chimpanzee (*Pan troglodytes*), NP001029079; Gorilla (*Gorilla gorilla*), XP004046638; Orangutan (*Pongo abelii*), XP002818825; Silvery Gibbon (*Hylobates moloch*), XP032015057; Northern White Cheek (NWC) Gibbon (*Nomascus leucogenys*), XP003271463; Green Monkey (*Chlorocebus sabaeus*), XP007959810; Gelada (*Theropithecus gelada*), XP025250382; Rhesus Macaque (*Macaca mulatta*), XP001098733; Drill (*Mandrillus leucophaeus*), XP011851075; Sooty Mangabey (*Cercocebus atys*), XP011933395. Accession numbers for HD5 ortholog sequences are: Human, NP066290; Chimpanzee, NP001012657; Gorilla, XP004046645; Orangutan, XP002818829; Silvery Gibbon, XP032015053; NWC Gibbon, XP003271465; Green Monkey, XP008017319; Gelada, XP025230170; Rhesus Macaque, AY859406; Drill, XP011851059; Sooty Mangabey, XP011890938.
(TIF)

S1 Table Bacterial Strains. Identity and source of bacterial strains used in this study.
(TIF)

S1 Video *S*. Typhimurium (5e6 CFU/ml) in buffer alone (tris-maleate pH 6.4), related to Fig 2B. (ZIP)

S2 Video Δ*fliC*Δ*fljB* STM *S*. Typhimurium (5e6 CFU/ml) in buffer alone (tris-maleate pH 6.4), related to Fig 2C. (ZIP)

S3 Video High-density *S*. Typhimurium (1e8 CFU/ml) in 20 μg/ml α-flagellin Ab, related to supplemental Fig 1. (ZIP)

S4 Video High-density *S*. Typhimurium (1e8 CFU/ml) in 10 μg/ml HD6, related to supplemental Fig 1. (ZIP)

S5 Video High-density *S*. Typhimurium (1e8 CFU/ml) in buffer alone (tris-maleate pH 6.4), related to supplemental Fig 1. (ZIP)

S6 Video *S*. Typhimurium (5e6 CFU/ml) in buffer alone (tris-maleate pH 6.4), related to Fig 2D. (ZIP)

S7 Video *S*. Typhimurium (5e6 CFU/ml) in 10 μg/ml HD6, related to Fig 2D. (ZIP)

S8 Video *S*. Typhimurium (5e6 CFU/ml) in in 20 μg/ml α-flagellin Ab, related to Fig 2D. (ZIP)

S9 Video *S*. Typhimurium (1e7 CFU/ml) in buffer alone (tris-maleate pH 6.4), related to Fig 3B. (ZIP)

S10 Video *S*. Typhimurium (1e7 CFU/ml) in 0.5 μg/ml HD6, related to Fig 3B. (ZIP)

S11 Video *S*. Typhimurium (1e7 CFU/ml) in 5 μg/ml HD6, related to Fig 3B. (ZIP)

S12 Video *S*. Typhimurium (1e7 CFU/ml) in 50 μg/ml HD6, related to Fig 3B. (ZIP)

S13 Video *S*. Typhimurium (1e7 CFU/ml) in buffer alone (tris-maleate pH 6.4), related to Fig 4D. (ZIP)

S14 Video *S*. Typhimurium (1e7 CFU/ml) in 0.5 μg/ml HD6^F2A^, related to Fig 4D. (ZIP)

S15 Video *S*. Typhimurium (1e7 CFU/ml) in 5 μg/ml HD6^F2A^, related to Fig 4D. (ZIP)

S16 Video *S*. Typhimurium (1e7 CFU/ml) in 50 μg/ml HD6^F2A^, related to Fig 4D. (ZIP)
