# Supplementary figures and images for: Flagella-driven motility is a target of human Paneth cell defensin activity
Source: PLoS Pathog. 2023 Feb 23;19(2):e1011200. doi: 10.1371/journal.ppat.1011200 (PMC9990921; doi:10.1371/journal.ppat.1011200)

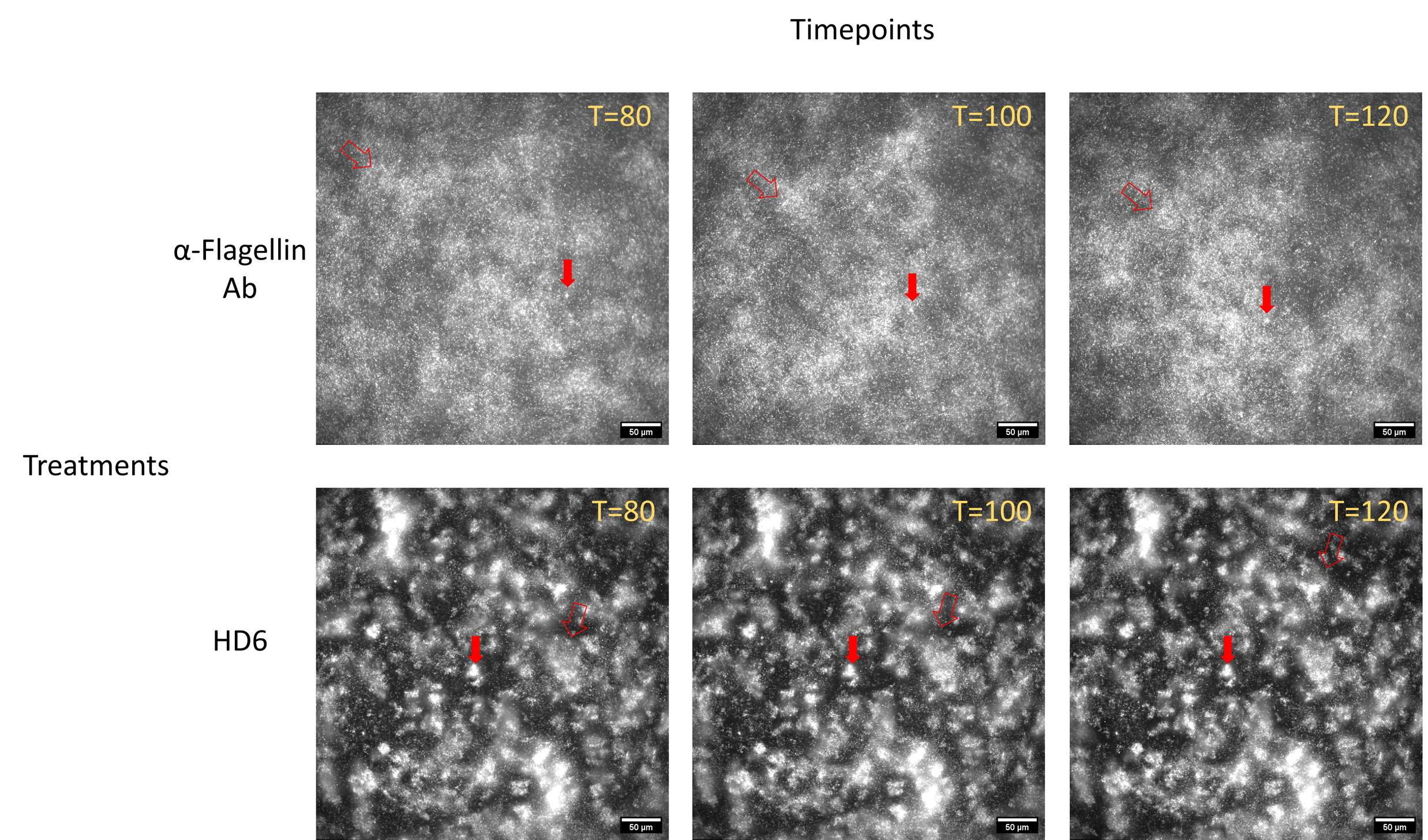

Supplement: S1 Fig — (A) Representative unmasked composite data depicting agglutinated S. Typhimurium at high densities (1e8 CFU/ml) in the presence of either 20 μg/ml α-Flagellin Ab (top row) or 10 μg/ml HD6. Areas of transiently higher bacterial density (open red arrow) and high-density bacterial aggregates (closed red arrow) are highlighted as examples of agglutination occurring. Data are representative of 2 independent experiments, each with technical replicates. Scale bar, 50 μm. (TIF) [file ppat.1011200.s001.tif]

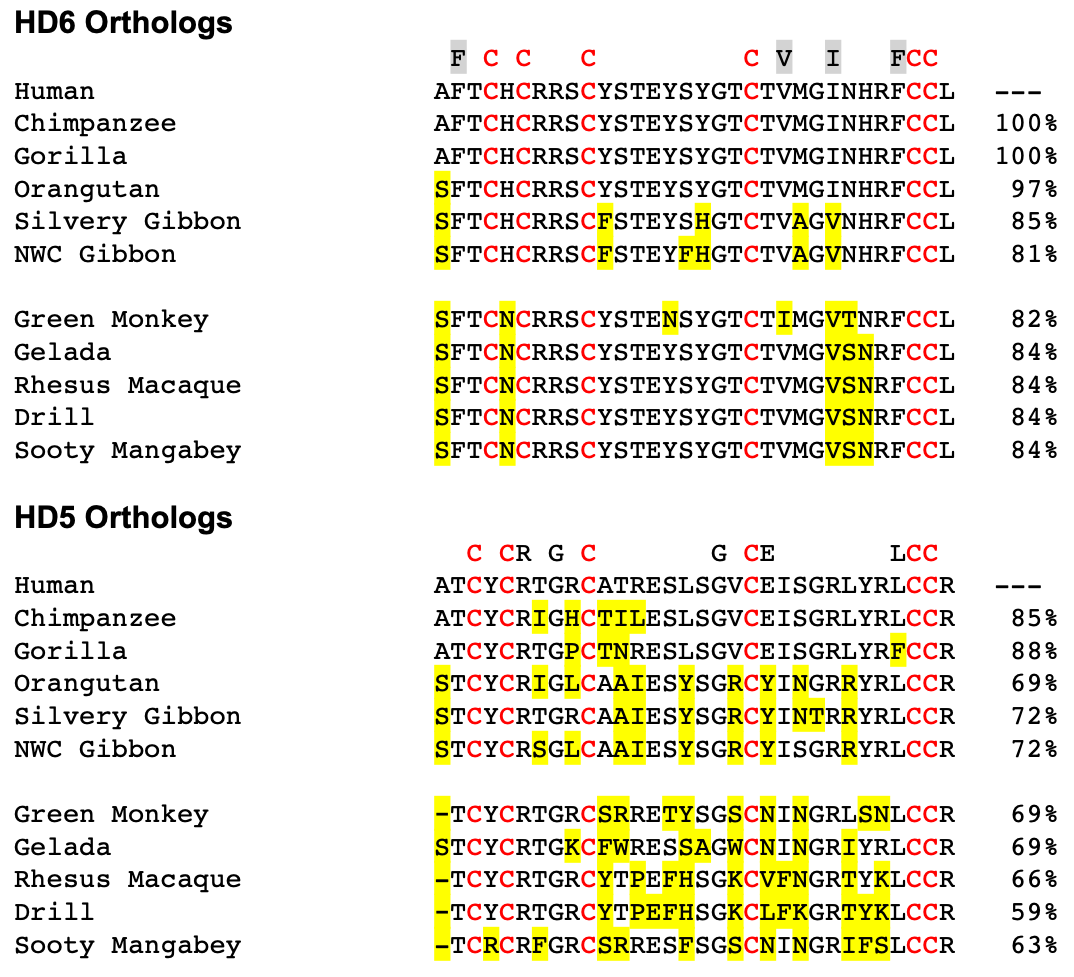

Supplement: S2 Fig — Highlighted with YELLOW are sequence variant residues for the HD5 and HD6 orthologs, respectively. Shown in RED are the cysteine residues that participate in three intramolecular disulfide bonds (Cys1-Cys6, Cys2-Cys4, Cys3-Cys5). Highlighted with GRAY in the top consensus row are the residues identified as key for structure-function of HD6 and HD5, respectively (Chairatana and Nolan 2014; Rajabi et al. 2012). The column at the left notes the percent of residue identity for HD6 and HD5 orthologs, respectively. Within each group of orthologs, the top cluster includes great apes and the bottom Old World monkeys. Accession numbers for HD6 ortholog sequences are: Human (Homo sapiens), AAC50382; Chimpanzee (Pan troglodytes), NP001029079; Gorilla (Gorilla gorilla), XP004046638; Orangutan (Pongo abelii), XP002818825; Silvery Gibbon (Hylobates moloch), XP032015057; Northern White Cheek (NWC) Gibbon (Nomascus leucogenys), XP003271463; Green Monkey (Chlorocebus sabaeus), XP007959810; Gelada (Theropithecus gelada), XP025250382; Rhesus Macaque (Macaca mulatta), XP001098733; Drill (Mandrillus leucophaeus), XP011851075; Sooty Mangabey (Cercocebus atys), XP011933395. Accession numbers for HD5 ortholog sequences are: Human, NP066290; Chimpanzee, NP001012657; Gorilla, XP004046645; Orangutan, XP002818829; Silvery Gibbon, XP032015053; NWC Gibbon, XP003271465; Green Monkey, XP008017319; Gelada, XP025230170; Rhesus Macaque, AY859406; Drill, XP011851059; Sooty Mangabey, XP011890938. (TIF) [file ppat.1011200.s002.tif]

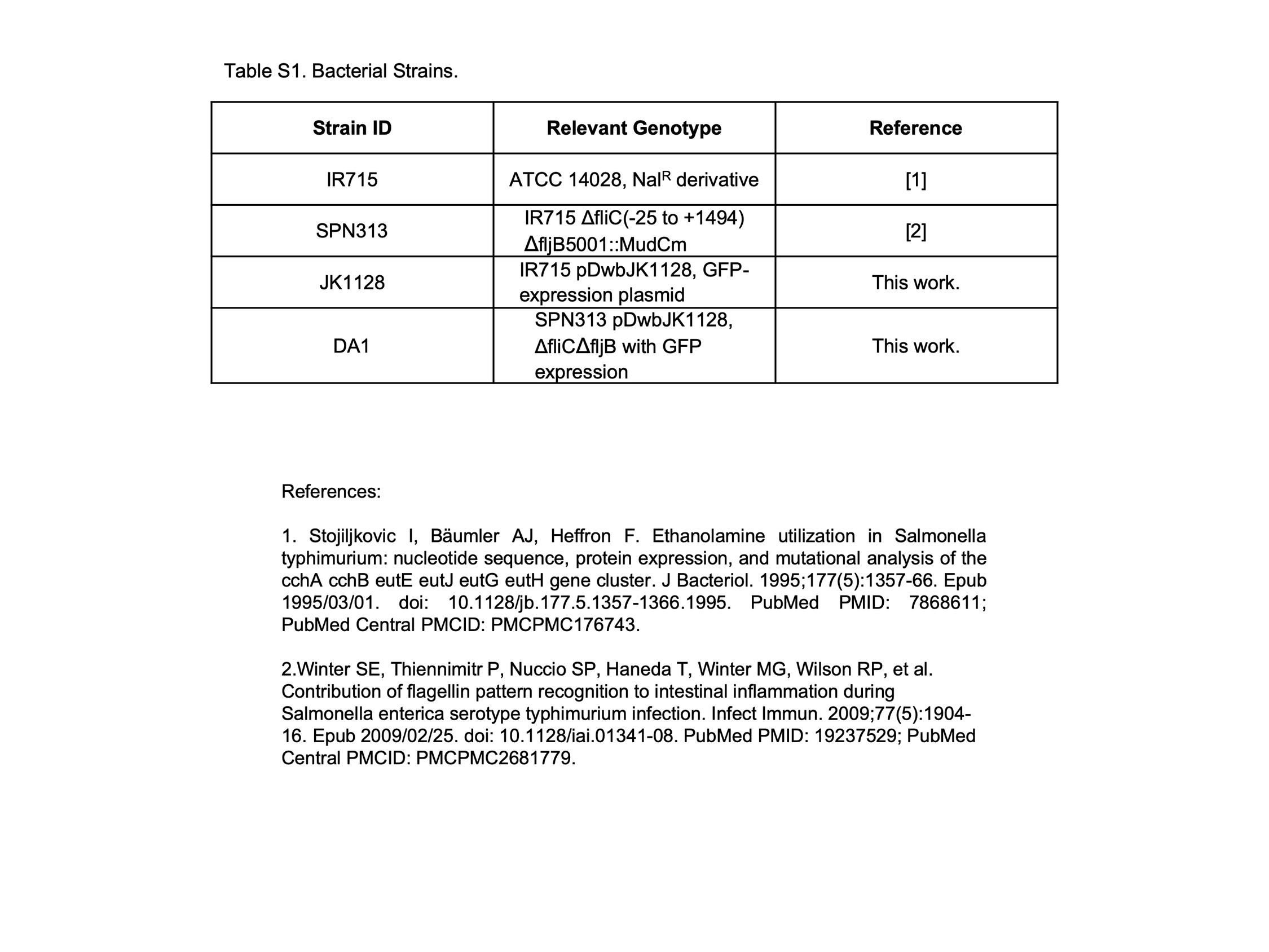

Supplement: S1 Table — Identity and source of bacterial strains used in this study. (TIF) [file ppat.1011200.s003.tif]
